# Intervention by a clinical pharmacist carried out at discharge of elderly patients admitted to the internal medicine department: influence on readmissions and costs

**DOI:** 10.1186/s12913-022-07582-6

**Published:** 2022-02-09

**Authors:** Andrea Lázaro Cebas, José Manuel Caro Teller, Carmen García Muñoz, Carlos González Gómez, José Miguel Ferrari Piquero, Carlos Lumbreras Bermejo, José Antonio Romero Garrido, Juana Benedí González

**Affiliations:** 1 Pharmacy Management Department. Dirección General de Asistencia Sanitaria, Servicio Murciano de Salud, Murcia, Spain; 2 Pharmacy Department. Hospital, Universitario 12 de Octubre, Madrid, Spain; 3 Servicio de Medicina Interna Hospital Universitario 12 de Octubre, Madrid, Spain; 4 Pharmacy Department Hospital, Universitario La Paz, Madrid, Spain; 5 Pharmacology Department. Facultad de Farmacia, Universidad Complutense, Madrid, Spain

**Keywords:** Pharmacists, Patient readmission, Aged, Polypharmacy, Cost analysis

## Abstract

**Background:**

Patient education on pharmacological treatment could reduce readmissions. Our objective was to carry out a pharmacist intervention focused on providing information about high-risk medications to chronic patients and to analyse its influence on readmissions and costs.

**Methods:**

A single-centre study with an intervention group and a retrospective control group was conducted. The intervention was carried out in all polymedicated patients ≥ 65 years who were admitted to internal medicine and signed the informed consent between June 2017 and February 2018. Patients discharged to nursing homes or long-term hospitals were excluded. The control group were all the patients who were admitted during the same months of 2014 who met the same inclusion criteria. The patients were classified according to the HOSPITAL score as having a low, intermediate, or high risk of potentially avoidable readmission. Outcome measures were 30-day readmission and cost data. To analyse the effect of the intervention on readmission, a logistic regression was performed.

**Results:**

The study included 589 patients (286 intervention group; 303 control group). The readmission rate decreased from 20.13% to 16.43% in the intervention group [OR = 0.760 95% CI (0.495–1.166); *p* = 0.209)]. The incremental cost for the intervention to prevent one readmission was €3,091.19, and the net cost saving was €1,301.26. In the intermediate- and high-risk groups, readmissions were reduced 10.91% and 10.00%, and the net cost savings were €3,3143.15 and €3,248.71, respectively.

**Conclusions:**

The pharmacist intervention achieved savings in the number of readmissions, and the net cost savings were greater in patients with intermediate and high risks of potentially avoidable readmission according to the HOSPITAL score.

## Background

The rise in life expectancy presents a significant challenge to health care systems in developed countries. As the population continues to age, the risk of chronic diseases associated with age and the demand for healthcare services are increasing [1].

Medication reconciliation and patient education about pharmacological treatment are recommended strategies for reducing medication errors during transitions of care [2–4]. However, when clinical pharmacists carry out these interventions in elderly patients, the results have been variable. The results of three meta-analyses indicate that medication reconciliation programmes performed by clinical pharmacists have reduced emergency department visits [2, 3, 5], medication-related adverse event visits [3, 5], medication errors [2], and readmissions [3].

Elderly polymedicated patients with chronic diseases are particularly vulnerable to hospital readmissions for any cause [4, 6], but the risk of readmission may vary within this patient group. In fact, according to different models that predict readmissions, such other variables as the number of previous admissions and the length of hospital stay can impact readmission, as can comorbidities, age, and polypharmacy [7]. Among the different predictive models of potentially avoidable readmissions published, the HOSPITAL score is a model that has been validated to identify patients admitted to medical services who have high, intermediate, or low risk of potentially avoidable readmission at the time of hospital discharge [8, 9]. In patients over 65 years, this model has good discriminatory power for patients with a high risk of potentially avoidable readmission as well as those with a high risk of general readmission [10].

In relation to polypharmacy, not all medications bring the same risk of hospital admission or readmission when errors associated with their use occur [4, 11]. For this reason, the members of the Institute for Safe Medication Practices in Spain published a list of high-alert medications for patients with chronic illnesses (HAMC list) to prioritize the implementation of effective interventions on medication safety [12].

Pharmacist interventions carried out in polymedicated elderly patients to improve pharmacotherapy and reduce readmission are heterogeneous in their nature and in their results. Furthermore, no studies have analysed whether interventions that aim to provide information about medications on the HAMC list to patients and caregivers could influence readmission or whether these interventions could be more effective in patients with a higher risk of potentially avoidable readmission.

The purpose of this study was to carry out a pharmacist intervention focused on providing information about medications on the HAMC list to polymedicated elderly patients admitted to the internal medicine ward. To analyse the impact of the intervention in this health service, a cost analysis was performed, and the influence on readmission rate was determined in the whole patient population and by subgroups of potentially avoidable readmission risk according to HOSPITAL score.

## Methods

### Study design

A single-centre study with an intervention group and a retrospective control group was carried out in a tertiary hospital where the internal medicine department staff treats patients in 4 independent hospitalization wards. The intervention was carried out for 9 months: June 2017 to February 2018.

### Participants

The study included elderly patients (≥ 65 years) who were polymedicated (5 or more chronic disease medications) and who had been admitted to one of the four internal medicine hospitalization wards during the study period. Patients with programmed admissions and patients who were discharged to nursing homes or long-term hospitals were excluded.

#### Intervention group

Patients admitted to one of the internal medicine hospitalization wards from June 2017 to February 2018 who met the inclusion criteria and signed the informed consent (IC) form were consecutively included. A clinical pharmacist who was integrated into the regular medical team carried out interventions on the patient’s treatment at different times during the healthcare process.

#### Control group

Patients admitted to the same hospitalization ward over the same months of 2014 who met the inclusion criteria were included in the control group. As the patients were recruited retrospectively, an IC form was not required.

### Data collection

The patients included in the study were divided into three groups according to HOSPITAL score: low risk of potentially avoidable readmission (0–4 points), intermediate risk (5–6 points), and high risk (≥ 7 points). The HOSPITAL score was calculated from seven parameters: haemoglobin (Hb) level < 12 g/dL in the last laboratory test prior to discharge (1 point), discharge from the oncology service (2 points), sodium < 135 mEq/L in the last laboratory test prior to discharge (1 point), procedures carried out during hospitalization according to the International Classification of Diseases (ICD-9) (1 point), urgent/emergent admission (1 point), number of hospitalizations during the previous year (0–1: 0 points, 2–5: 2 points, > 5: 5 points), and length of stay > 5 days (2 points) [8].

The following variables were collected from both patient groups through their electronic medical records: demographics (sex and age); degree of dependence (independent or low dependence, moderate dependence, and severe or total dependence); comorbidities (cognitive impairment, heart failure, stroke, chronic pulmonary disease, chronic liver disease, chronic kidney disease (CKD), and diabetes); diagnosis on admission and readmission classified according to the following categories: heart disease, respiratory disease, kidney disease, gastrointestinal disease, neurological disease, liver or biliary disease, endocrine system (diabetes or electrolyte abnormalities), infection, neoplasia (solid or haematological), and other; number of chronic medications; and number of medications from the HAMC list prescribed at discharge. From the HAMC list, the following groups of medications were considered: antiplatelets, oral anticoagulants, nonsteroidal anti-inflammatory drugs (NSAIDs), central nervous system depressants (benzodiazepines and opioids), antiarrhythmic agents (β-adrenergic blockers, digoxin, and amiodarone), insulin, oral hypoglycaemic agents, and diuretics (loop diuretics) [12]. In addition, based on a consensus between internal medicine and pharmacy departments, the following drugs and therapeutic groups were included: diltiazem and verapamil (antiarrhythmic agents), thiazides (diuretics), and renin-angiotensin system inhibitors (angiotensin-converting enzyme inhibitors (ACEIs) or angiotensin receptor blockers (ARBs)).

### Pharmacist intervention

At admission: Interview with the patient and/or main caregiver and medication reconciliation.

During hospitalization: Pharmacotherapeutic follow-up (validation of the prescriptions).

On discharge: Interview with the patient and/or main caregiver, including an educational intervention regarding postdischarge treatment (written treatment plan and written information about HAMC): information on changed dose, medication initiations or discontinuations and the detection and management of possible adverse events associated with the different HAMC.

At home (phone call follow-up): A phone call at 7 and 21 days after discharge, with completion of a questionnaire to measure the degree of knowledge of patients and/or their main caregivers regarding HAMC.

The questionnaire used to measure the patient’s knowledge of HAMC was a validated questionnaire of 11 questions. This questionnaire assesses knowledge in four dimensions: therapeutic objective (therapeutic indication and effectiveness), process of use (dosage, regimen, administration, and duration of treatment), safety (adverse reactions, precautions, contraindications, and interactions), and storage [13]. The answers to the questions were categorized by the interviewing pharmacist as “is aware” or “is not aware” according to the information provided by the patient or caregiver.

### Outcome measures

Readmission was defined as all-cause 30-day hospital readmission. The readmission rate was determined in the whole patient population and in the 3 different categories of potentially avoidable readmission risk defined by the HOSPITAL score.

To analyse the cost of the intervention, the absolute risk reduction (ARR) of the readmission rate from the control to the intervention group and the number needed to treat (NNT) were calculated. The costs considered to do the analysis were a median cost per admission in patients older than 65 years of €4,392.45 [14] and a median annual clinical pharmacist salary of €45,749.58 [15]. The patient population who could be recipients of the intervention over 1 year was estimated to be 400.

The cost analysis was performed for all patients and for the patients in the 3 different categories of potentially avoidable readmission risk defined by the HOSPITAL score.

### Statistical Analysis

A descriptive analysis of baseline characteristics was performed on the patients in the control and intervention groups. Categorical and quantitative variables were tested by the χ^2^ test or Fisher's exact test, and continuous variables were assessed using the nonparametric Mann–Whitney-Wilcoxon U tests, as stipulated by the Shapiro–Wilk normality test. Categorical variables are presented as number and percentage, and continuous variables are presented as median and interquartile range (IQR).

A binary logistic regression was performed to analyse the effect of the intervention on readmission. In the multivariate analysis, all variables with statistically significant differences (*p* < 0.05) between the control and intervention groups were included. The results are expressed as *odds ratio* (OR) with 95% confidence interval (95% CI).

Statistical analysis was carried out using Stata® 16 statistical software (StataCorp. 2019. Stata Statistical Software: Release 16. College Station, TX: StataCorp LLC). A significance level of 5% was used in this study.

## Results

**Fig. 1:**
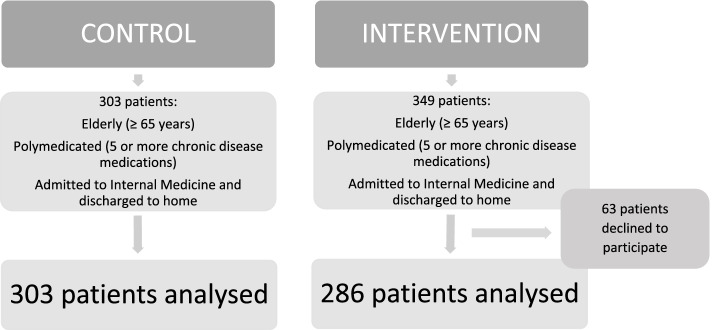
Flow chart

A total of 589 patients were included in the present study: 286 patients in the intervention group and 303 in the control group (Fig. 1).

The baseline characteristics of both groups are listed in Table 1. All variables measured were comparable (*p* > 0.05) except age (higher in the intervention group) and CKD (more prevalent in the intervention group).

**Table 1:** Patient characteristics at baseline

	Total *n* = 589	Control *n* = 303	Intervention *n* = 286	*p* value
	No. (%)	No. (%)	No. (%)	
Sex (male)	259 (43.97)	131 (43.23)	128 (44.76)	0.710
Age, years (median, IQR)	83 [78–87]	83 [77–86]	84 [79–88]	0.0044
Length of stay, days (median, IQR)	8 [5-12]	8 [5-12]	7 [5-11]	0.055
No. of medications at discharge (median, IQR)	9 [7-11]	9 [7-11]	9 [7-11]	0.3320
No. of HAMC list drugs at discharge (median IQR)	4 [3-5]	4 [3-4]	4 [3-5]	0.5207
**Degree of dependence**				0.199
Independent or low dependence	361 (61.29)	179 (59.08)	182 (63.64)	
Moderate dependence	168 (28.52)	96 (31.68)	72 (25.17)	
Severe or total dependence	60 (10.19)	28 (9.24)	32 (11.19)	
**Comorbidities**				
No. of comorbidities (median, IQR)	2 [1-3]	2 [1,2]	2 [1-3]	0.3490
Cognitive impairment	118 (20.03)	55 (18.15)	63 (22.03)	0.240
Heart failure	233 (39.56)	113 (37.29)	120 (41.96)	0.247
Chronic pulmonary disease	225 (38.20)	117 (38.61)	108 (37.76)	0.832
Chronic kidney disease	141 (23.94)	61 (20.13)	80 (27.97)	0.026
Chronic liver disease	30 (5.09)	18 (5.94)	12 (4.20)	0.336
Diabetes	241 (40.92)	128 (42.24)	113 (39.51)	0.500
Stroke	77 (13.07)	41 (13.53)	36 (12.59)	0.734
**Primary admission diagnosis**				0.189
Heart disease	187 (31.75)	103 (33.99)	84 (29.37)	
Respiratory disease	57 (9.68)	30 (9.90)	27 (9.44)	
Kidney disease	5 (0.85)	4 (1.32)	1 (0.35)	
Gastrointestinal disease	28 (4.75)	17 (5.61)	11 (3.85)	
Neurological disease	23 (3.90)	14 (4.62)	9 (3.15)	
Liver or biliary disease	14 (2.38)	5 (1.65)	9 (3.15)	
Endocrine system (diabetes or electrolyte abnormalities)	10 (1.70)	5 (1.65)	5 (1.75)	
Infection	197 (33.45)	86 (28.38)	111 (38.81)	
Neoplasia (solid or haematological)	15 (2.55)	7 (2.31)	8 (2.80)	
Others	53 (9.00)	32 (10.56)	21 (7.34)	
**Risk of potentially avoidable readmission (HOSPITAL score)**				0.806
Low	362 (61.46)	185 (61.06)	177 (61.89)	
Intermediate	189 (32.09)	100 (33.00)	89 (31.12)	
High	38 (6.45)	18 (5.94)	20 (6.99)	

*HAMC* high-alert medications for patients with chronic illnesses

Of the 572 follow-up phone calls that could be made to the 286 patients in the intervention group, 491 were carried out. Two hundred sixty-three (53.56%) were made 7 days after discharge, and 228 (46.44%) were made on day 21. All possible phone calls could not be made because of patient readmission (60.73%, *n* = 50) and the inability to contact the patient or caregiver (38.27%, *n* = 31).

The number of phone calls answered by the patients was 192 (39.10%). In 82.81% (*n* = 159) of them, the patients reported independently taking and organizing their medication without assistance. The other phone calls (60.90%, *n* = 299) were answered by caregivers who prepared and/or administered the patient treatment. The distribution of the patient’s knowledge in each of the dimensions of the questionnaire by HAMC group is shown in Table 2. No questions were asked about NSAIDs, as none of the patients were on NSAID treatment.

**Table 2:** Patients’ distribution of knowledge in each of the dimensions of the questionnaire by HAMC group

	Antiplatelets (*n* = 25)	Antiarrhythmic agents (*n* = 46)	Oral anticoagulants (*n* = 99)	Oral hypoglycaemic agents (*n* = 30)	ACEIs or ARBs (*n* = 67)	CNS depressants (*n* = 51)	Diuretics (*n* = 131)	Insulin (*n* = 42)
	Is aware, No. (%)	Is aware, No. (%)	Is aware, No. (%)	Is aware, No. (%)	Is aware, No. (%)	Is aware, No. (%)	Is aware, No. (%)	Is aware, No. (%)
Therapeutic objective								
What is the therapeutic indication of this medication?	20 (80.00)	34 (73.91)	85 (85.86)	28 (93.33)	47 (70.15)	48 (94.12)	111 (84.73)	42 (100.00)
How do you know if the medication is effective?	7 (28.00)	28 (60.87)	58 (58.59)	15 (50.00)	44 (65.67)	40 (78.43)	110 (83.97)	40 (95.24)
Process of use								
What is the dosage of this medication that you must take?	25 (100.00)	40 (86.96)	93 (93.94)	30 (100.00)	62 (92.54)	48 (94.12)	124 (94.66)	42 (100.00)
What is the regimen for taking this medication?	25 (100.00)	42 (91.30)	94 (94.95)	30 (100.00)	63 (94.03)	47 (92.16)	125 (95.42)	42 (100.00)
What is the treatment duration for this medication?	25 (100.00)	42 (91.30)	94 (94.95)	30 (100.00)	63 (94.03)	47 (92.16)	126 (96.18)	42 (100.00)
How should you administer this medication?	25 (100.00)	42 (91.30)	94 (94.95)	29 (96.67)	63 (94.03)	47 (92.16)	126 (96.18)	42 (100.00)
Safety								
Should you take any precautions when you take this medication? Which ones?	10 (40.00)	11 (23.91)	60 (60.61)	3 (10.00)	5 (7.46)	11 (21.57)	39 (29.77)	16 (38.10)
Do you know the adverse reactions of this medication?	10 (40.00)	5 (10.87)	39 (39.39)	4 (13.33)	5 (7.46)	8 (15.69)	7 (5.34)	15 (35.71)
What special situations or health problems contraindicate the use of this medication?	2 (8.00)	2 (4.35)	6 (6.06)	2 (6.67)	2 (2.99)	1 (1.96)	3 (2.29)	6 (14.29)
Which other medications or food should you avoid while you are taking this medication?	1 (4.00)	1 (2.17)	6 (6.06)	2 (6.67)	1 (1.49)	1 (1.96)	1 (0.76)	0 (0.00)
Storage								
Where should you store this medication?	25 (100.00)	42 (91.30)	86 (86.87)	29 (96.67)	67 (100.00)	44 (86.27)	119 (90.84)	40 (95.24)

*HAMC* high-alert medications for patients with chronic illnesses, *ACEIs* angiotensin-converting enzyme inhibitors, *ARBs* angiotensin receptor blockers, *CNS* central nervous system

The 30-day readmission rate was 20.13% (*n* = 61) in the control group and 16.43% (*n* = 47) in the intervention group [OR = 0.780 95% CI (0.512–1.188); *p* = 0.247)]. The effect of the intervention was not modified in the multivariate analysis when it was adjusted by age and CKD [OR = 0.760 95% CI (0.495–1.166); *p* = 0.209)] (Table 3).

**Table 3:** Estimation of the pharmacist intervention effect on readmission after adjustment for age and chronic kidney disease

	OR	95% CI	*p* value
Pharmacist intervention	0.760	0.495–1.166	0.209
Age, years	0.977	0.948–1.007	0.136
Chronic kidney disease	1.891	1.193–2.998	0.007

The most frequent diagnoses at readmission in the intervention and control groups were those related to heart disease (41.94% control group vs. 28.26% intervention group) and infection (27.42% control group vs. 32.61% control group). Readmissions related to heart disease were 13.68 percentage points less common in the intervention group than the control group.

In the subgroup of patients with a low risk of potentially avoidable readmission, the 30-day readmission rate was similar between the control and intervention groups (11.89% vs 12.43%). In the subgroups of patients with intermediate and high risk of potentially avoidable readmission, reductions in readmission rates were observed in the intervention group (Table 4). All the patients from the intervention and control groups, including those in the intervention group who did not complete the phone call follow-up, were included in the analysis. The cost analysis was performed in the whole patient sample and in the patients with intermediate or high risk of potentially avoidable readmission. The net cost savings per readmission adverted were €1,301.26 in the whole set of patients, €3,343.15 in the group with intermediate risk of potentially avoidable readmission and €3,248.71 in the high-risk group. A summary of the cost analysis is given in Table 4.

**Table 4:** Cost analysis

	Total		Intermediate risk of potentially avoidable readmission		High risk of potentially avoidable readmission	
	Control (*n* = 303)	Intervention (*n* = 286)	Control (*n* = 100)	Intervention (*n* = 89)	Control (*n* = 18)	Intervention (*n* = 20)
30-day readmissions, No. (%)	61 (20.13)	47 (16.43)	30 (30.00)	17 (19.10)	9 (50.00)	8 (40.00)
ARR of 30-day readmission (%)	3.70		10.90		10.00	
NNT	27		9		10	
Incremental cost for the intervention to prevent one readmission ^a^ (€)	3,091.19		1,049.30		1,143.74	
Cost saving ^b^ (€)	1,301.26		3,343.15		3,248.71	

*ARR* absolute risk reduction *NNT* number needed to treat

^a^NNT × Cost of the intervention per patient [€45,749.58 (median annual clinical pharmacist salary)/400 (patient population that could be recipients of the intervention over 1 year]

^b^Incremental cost for the intervention to prevent one readmission – Cost per admission (€4,392.45)

## Discussion

In this study, the pharmacist intervention achieved cost savings, and the absolute reduction in readmission rate was approximately 4%. These results were in accordance with previous publications [16–21]. In some published studies, pharmacist interventions did not significantly decrease readmission [16, 18–22], while other studies that evaluated the effect of this type of intervention found statistically significant effects [23–26].

Our pharmacist intervention was focused on providing information on medications with a high risk of adverse events associated with erroneous use in elderly patients with chronic diseases [12]. The degree of patient knowledge of HAMC varied widely between the different questionnaire dimensions. In the dimension related to the process of use, a mean of more than 95.00% of patients or caregivers knew the answers, whereas in the safety block of questions, only a mean of 13.00% were aware of the relevant information. One explanation might be that at the time of hospital discharge, both patients and caregivers tend to focus on basic treatment information (dosage, regimen, administration, and duration of treatment), but knowledge of medication safety is part of an advanced understanding of medication management, so the time of hospital discharge might not be the best time to share this information with patients and caregivers.

According to published studies, pharmacist interventions that focus on increasing the knowledge of elderly patients with chronic diseases on high-risk medications had better results when they were combined with other measures to improve care coordination and comprehensive patient care [19, 23–26]. In our study, the pharmacists could not collaborate with care coordinators, primary care physicians or community pharmacists, so the HAMC safety information was shared at hospital discharge instead of at a time more conducive to effective communication, which would have enabled both patient and caregiver to better understand the information.

Readmission due to heart disease was the diagnosis that was most reduced by the intervention. This may be because, within the readmission diagnoses, decompensation of heart failure is the condition that best lends itself to prevention through proper knowledge and management of the different drugs on the HAMC list (mainly diuretics but also ACEIs, ARBs, and antiarrhythmic agents) [27].

The net cost saving of the intervention per readmission adverted was €1,301.26. Extrapolated to the United States, considering a median cost per admission in patients older than 65 years of $14,420 [28] and a median annual clinical pharmacist salary of $128,090 [29], the net cost saving of the intervention per readmission adverted would be $5,473.93. Given that the patients included in this study suffered from chronic and multiple diseases, readmissions are relevant not only because of their health care costs but also because of their impact on the patient’s functional status, quality of life, morbidity, and mortality [30].

The group of patients selected for the intervention in this study were already at high risk of readmission [4, 6]. The HOSPITAL predictive model is a valid and easy-to-apply tool for selecting, within this high-risk patient group, those who could benefit more from such a pharmacist intervention.

Several limitations of this study should be noted. Due to the limited time the pharmacist intervention was carried out, too few patients were included in the study to demonstrate the benefit of the intervention on readmission with adequate power. Even so, the percentage readmission reduction was comparable to that in previous studies. Other limitations were the single-centre nature and the use of a retrospective control group. Despite the use of a retrospective control group, patient characteristics at baseline between the control and intervention groups were comparable.

The study had several strengths. First, the intervention was focused on a target population recognized by healthcare institutions in their attempt to reduce readmissions. Second, the intervention was focused on improving patient safety by providing information about medications with a high risk of producing adverse events in chronic patients.

## Conclusions

The pharmacist intervention implemented in this study achieved cost savings and reduced the frequency of readmissions. The net cost savings of the intervention were the best in the selection of patients with intermediate and high risk of potentially avoidable readmission. The HOSPITAL score has been demonstrated to be a valid and easy-to-apply tool for selecting patients who could benefit the most from such pharmacist interventions.

## Data Availability

Data will be available on request to the corresponding author due to privacy/ethical restrictions. E-mail address: andrea.lazaro@carm.es.

## Abbreviations

HAMC: High-alert medications for patients with chronic illnesses
IC: Informed consent
ICD: International Classification of Diseases
CKD: Chronic kidney disease
NSAIDs: Nonsteroidal anti-inflammatory drugs
ACEIs: Angiotensin-converting enzyme inhibitors
ARBs: Angiotensin receptor blockers
ARR: Absolute risk reduction
NNT: Number needed to treat
